# Complete genome sequence of fluoroacetate-degrading *Caballeronia* sp. S22 strain (DSM 8341) as a reference resource for investigations of microbial defluorination

**DOI:** 10.1128/mra.00812-24

**Published:** 2024-12-27

**Authors:** Catherine Badel, Enrico Bocconetti, Radi Khodr, Claire Husser, Michael Ryckelynck, Stéphane Vuilleumier

**Affiliations:** 1 Génétique Moléculaire, Génomique, Microbiologie, UMR 7156 CNRS, Université de Strasbourg, Strasbourg, France; 2 Architecture et Réactivité de l'ARN, UPR 9002 CNRS, Université de Strasbourg, Strasbourg, France; Rochester Institute of Technology, Rochester, New York, USA

**Keywords:** defluorination, fluoride, genome sequence, PFAS, dehalogenation, fluorinated compounds

## Abstract

A complete genome sequence of *Caballeronia* sp. strain S22 capable of growing with fluoroacetate as the sole source of carbon and energy was obtained by PacBio technology. It consists of seven circular replicons totaling 9,367 kb, with a gene cluster involved in fluoroacetate utilization on its smallest 172 kb plasmid.

## ANNOUNCEMENT

Among the 8,400 halogenated compounds now known to be produced naturally on Earth (1), very few natural fluorinated compounds have been reported (2). Extensive human use of poly- and perfluoroalkane substances (PFAS) has increased the number of fluorochemicals a millionfold (3). The toxicity of PFAS to humans and ecosystems is fueling interest in the discovery of enzymes capable of degrading fluorinated chemicals (4, 5). We report the genome sequence of *Caballeronia* sp. S22, a well-investigated model strain for defluorination metabolism isolated from Australian soil with fluoroacetate as the sole carbon source (6–9).

Genomic DNA of the strain obtained from the DSMZ culture collection (DSM 8341) was extracted from an R2A culture grown at 30°C using the MasterPure Complete DNA Purification Kit (LuciGen) according to the manufacturer’s instructions. An SMRTbell express template prep kit 2.0 was used for library preparation from sheared DNA fragments of approximately 10 kb on average with g-tubes (Covaris) and AMPure PB beads following the Pacific Biosciences (PacBio) protocol. Sequencing was performed with an SMRTBell polymerase complex obtained using Binding kit 2.2, primer v5, an 8M SMRTcell, and a sequencing plate 2.0 on a PacBio Sequel II sequencer. Default parameters were used with all software unless otherwise specified. Raw sequencing data (CCS reads) were treated using smrttools10.1.0.119588 (3 minimum passes for “ccs”; split-bam-named, ccs and peek-guess for “lima”), yielding 680,112 reads (mean 8,620, maximum 28,236, total 5,862,279,966 nucleotides; PacBio N50 value 9,049).

Genome assembly and circularization of contigs were performed using the trycycler pipeline (programs “sample”, “cluster”, “reconcile”, “msa”, “partition”, and “consensus”) (10). Reads were first subsampled three times independently with “sample” using m parameter set at 5, 10, and 25, yielding 23.5-, 41.9-, and 88.3-fold coverage (expected 7 Mb genome size). Assemblers Flye (11), Canu (12), Raven (13), and Minimap2/Miniasm (14) were applied to each subsample using default parameters. Assemblies with the lowest number of contigs were selected and clusters of non-singleton contigs were obtained using “cluster”. The program “reconcile” was used for trimming and circularization through a systematic alignment of contigs from different assemblies and read subsets. This resulted in seven circularized contig clusters with a start position defined by the program. Programs “msa”, “partition”, and “consensus” yielded a high-quality assembly for each replicon (Table 1).

**TABLE 1:** Genome characteristics of *Caballeronia* sp. strain S22 and homologous replicons in the three complete assembled reference genomes of the *Caballeronia* genus at NCBI^*a*^

Strain-S22 replicon	Size (bp)	Coverage (fold)^*b*^	GC (%)	CDS	rRNA operons	tRNAs	*C. insecticola^c^* RPE64 (bp)	*C. zhejiangensis^d^* A33_M4_a (bp)	*C. grimmiae^e^* Lep1A1 (bp)
									
a	3,233,214	694	62.8	3,174	4	56	3,013,410 (80%)	3,035,511 (81%)	2,870,354 (77%)
(OZ180035.1)							(NC_021287.1^*f*^)	(NZ_CPO84283.1^*f*^)	(NZ_CP084271.1^*f*^)
b	1,822,652	691	62.6	1,862	1	4	1,465,356 (64%)	1,407,959 (63%)	1,226,197 (49%)
(OZ180036.1)							(NC_021294.1^*f*^)	(NZ_CPO84284.1^*f*^)	(NZ_CP0842272.1^*f*^)
c	1,447,788	686	62.9	1,340	1	2			
(OZ180039.1)									
d	1,169,233	662	63.5	1,126	0	0	1,275,199 (67%)	1,094,832 (64%)	
(OZ180038.1)							(NC_021289.1^*g*^)	(NZ_CPO84285.1^*g*^)	
e	892,580	622	60.7	1,069	0	1			
(OZ180040.1)									
f	630,138	669	62.2	612	0	1	900,830 (46%)	759,539 (57%)	
(OZ180037.1)							(NC_021288.1^*f*^)	(NZ_CPO84286.1^*f*^)	
g	171,990	1122	59.6	213	0	0			
(OZ180041.1)									

^*a*^ Replicon accession numbers are given between brackets. Percentage synteny between replicons (>40%), as identified by MicroScope (15), is indicated between square brackets.

^*b*^ Estimated as (number of reads) * N50 / (replicon size).

^*c*^ The *C. insecticola* genome (6,964 kb, completeness 99.0%, contamination 1.3%) has one additional 309,692 bp plasmid (NC_021295.1).

^*d*^ The *C. zhejiangensis* genome (7,412 kb, completeness 99.9%, contamination 1.0%) has three additional plasmids of 666,467 bp (NZ_CP084287.1), 439,699 bp (NZ_CP084288.1, 1 rRNA operon), and 8,158 bp (NZ_CPO289.1).

^*e*^ The *C. grimmiae* genome (6,651 kb, completeness 99.5%, contamination 0.9%) has one additional chromosome of 652,162 bp (NZ_CP084274.1) and five additional plasmids of 916,501 (NZ_CP084273.1), 424,980 bp (NZ_CP0084275.1), 363,975 bp (NZ_CP0084276.1), 131,480 bp (NZ_CP0084277.1), and 65,095 bp (NZ_CP0084278.1).

^*f*^ Reported as a chromosome. The number of rRNA operons is the same as in *Caballeronia* sp. strain S22.

^*g*^ Reported as a plasmid without rRNA operons.

**Fig 1:**
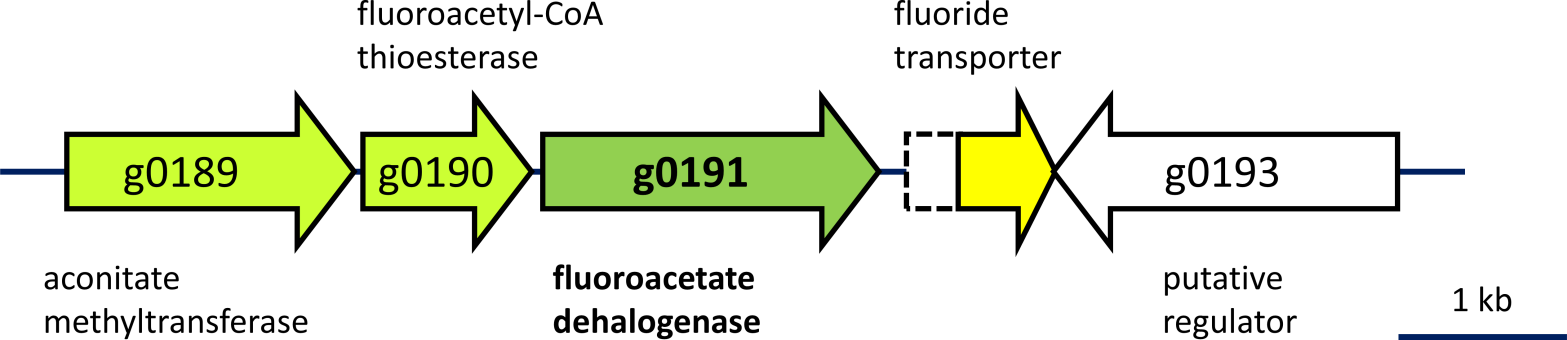
Fluoroacetate dehalogenase gene cluster. CABS22_g0191 encodes a protein with high sequence identity to experimentally characterized fluoroacetate dehalogenases (17). It is flanked by genes encoding known determinants of resistance to fluoroacetate. Aconitate methyltransferase (CABS22_g0189) allows esterification of fluorinated aconitate produced from fluoroacetate by enzymes of the TCA cycle (18), fluoroacetyl-CoA thioesterase (CABS22_g0190) selectively hydrolyses fluoroacetyl-CoA preventing its entry in the TCA cycle (19), and the fluoride exporter CrcB (CABS22_g0192) excretes toxic fluoride produced by dehalogenation of fluoroacetate (20). The DNA sequence of the fluoride exporter gene potentially encodes a larger fluoride transporter (hyphenated extension at the 5′-end of CABS22_g0192) upon −1 translational frameshifting (the frameshift sequence was checked by Sanger sequencing).

The replicons of strain S22 show marked differences with reference *Caballeronia* genomes (Table 1). The quality of the genome was verified with CheckM (V1.2.3; 64 genomes, 769 lineage-specific markers; completeness 100%, contamination 2.2%, 11 duplicated markers) (16). Genome annotation was performed using the MicroScope platform (15). Several CDS show similarity to functional fluoroacetate dehalogenases, and one gene cluster on replicon g contains one such fluoroacetate dehalogenase homolog surrounded by genes associated with resistance to fluoroacetate toxicity (Fig. 1). The genome sequence of *Caballeronia* sp. strain S22 will prove useful in future investigations of defluorination, toxicity of fluorinated compounds and of fluoride released by defluorination, and bacterial growth with fluorinated chemicals.

## Data Availability

The project (Bioproject PRJEB74306) was deposited in ENA, genome assembly GCA_964261745, accession numbers OZ180035–OZ180041 as listed in Table 1.
